# Oxalic Acid From *Sesbania rostrata* Seed Exudates Mediates the Chemotactic Response of *Azorhizobium caulinodans* ORS571 Using Multiple Strategies

**DOI:** 10.3389/fmicb.2019.02727

**Published:** 2019-12-03

**Authors:** Xiaolin Liu, Kaiye Zhang, Yanan Liu, Zhihong Xie, Chengsheng Zhang

**Affiliations:** ^1^Key Laboratory of Coastal Biology and Bioresource Utilization, Yantai Institute of Coastal Zone Research, Chinese Academy of Sciences, Yantai, China; ^2^College of Resources and Environment, University of Chinese Academy of Sciences, Beijing, China; ^3^College of Agriculture, Shanxi Agricultural University, Taigu, China; ^4^Center for Ocean Mega-Science, Chinese Academy of Sciences, Qingdao, China; ^5^Marine Agriculture Research Center, Tobacco Research Institute of Chinese Academy of Agricultural Sciences, Qingdao, China

**Keywords:** organic acids, chemotaxis, plant exudates, colonization, oxalic acid

## Abstract

Chemotaxis toward seed exudates is important in the establishment of microbe–plant associations. The objective of this work was to explore whether organic acids from the seed exudates of *Sesbania rostrata* play a role in recruiting *Azorhizobium caulinodans* ORS571 in the plant rhizosphere. High-performance liquid chromatography (HPLC) was used to analyze the organic acid content in seed exudates of *S. rostrata* and to further determine their roles in *A. caulinodans* growth and chemotactic response. Succinic, acetic, citric, oxalic, and lactic acids were the most abundant, and, except for oxalic acid, they could support *A. caulinodans* growth as the sole carbon source. TlpA1, a transmembrane chemoreceptor, was found to be involved in the chemotactic response to these organic acids. Oxalic acid played a direct role in the chemotactic response, but it also played an indirect role by promoting or inhibiting the chemotactic response toward other chemoeffectors. Furthermore, the indirect role of oxalic acid on other chemoeffectors was concentration-dependent. The effect of oxalic acid at different concentrations on host root colonization was also determined. By using different strategies, oxalic acid appears to play a major role in the early steps of the association of *A. caulinodans* and its host plant.

## Introduction

Bacterial chemotaxis towards plants plays a major role in the colonization of the root surface, and chemotaxis signaling appears as an early event in communication between plants and rhizobia (Nelson, 2004). The composition and concentration of seed exudates differ with plant species; they usually contain organic acids, amino acids, sugars, flavonoids, and proteins, which are potential chemoeffectors to bacteria. In canonical chemotaxis systems, binding of ligands/chemoeffectors to chemoreceptors, encoded by *mcp* genes, affects CheA, a histidine kinase, autophosphorylation (Szurmant and Ordal, 2004). The response regulator CheY, which can be phosphorylated by CheA, diffuses in the cytoplasm and ultimately interacts with flagellar motor proteins, triggering the changes in movement (Eisenbach, 1996; Szurmant and Ordal, 2004).

Production of chemo-attractants to soil bacteria by plant seeds and roots is a common feature that can affect the association between plants and microbes via different strategies. In particular, organic acids promote bacterial root adsorption, swarming motility, and plant colonization (Hida et al., 2015; Li et al., 2017). For example, clover exudates promote plant root colonization of *Rhizobium leguminosarum* bv. Trifolii (Janczarek and Skorupska, 2011), and exudates from Alfalfa stimulate adsorption of *Rhizobium meliloti* to the root surface (Wall and Favelukes, 1991). The quantity of exudates increases with the growth of plants, and their composition may differ at different plant ages (Jones et al., 2004).

Organic acids, including succinic, citric, malic, and lactic acids are predominant components in bean, tobacco, maize, tomato, cucumber, and sweet pepper exudates (Berendsen et al., 2012; Neal et al., 2012; Li et al., 2017; Martins et al., 2017) and have multiple effects on bacteria and plants. (1) As a growth substrate. For example, oxalic acid, in *Ralstonia eutropha* and *Methylobacterium extorquens*, can serve as the sole carbon source and energy to support growth (Sahin, 2003). Citric acid, succinic acid, and aromatic organic acids produced by plant exudates are preferentially consumed by rhizosphere bacteria (Kuiper et al., 2002; Zhalnina et al., 2018). (2) As an inducer of the chemotactic response. In particular, malic, fumaric, citric, cinnamic, and succinic acids can enhance cognate *mcp* gene expression levels (Tan et al., 2013; Li et al., 2017). For example, acetate, in *Pseudomonas putida*, increases the transcript level of its cognate chemoreceptor by up to 22-fold (Lopez-Farfan et al., 2017). (3) By affecting bacterial plant surface colonization and biofilm formation. For example, malic acid in the root exudates of banana and tomato have been shown to favor root colonization by *Bacillus amyloliquefaciens* (Tan et al., 2013; Yuan et al., 2015). (4) As protection against metal stress (Kamilova et al., 2006) and reactive oxygen species (ROS) (Haichar et al., 2014). For example, citric acid and oxalic acid have been shown to scavenge metals for soil fungi (Van Hees et al., 2006). *Pseudomonas fluorescens* produces oxalate to prevent aluminum stress (Hamel et al., 1999), and in *Caulobacter crescentus*, organic acids prevent nickel toxicity (Jain and Chen, 2018). (5) In addition, oxalic acid can function as a signal molecule and affect competition and pathogenicity in the fungi–bacteria interaction (Rudnick et al., 2015; Ul Haq et al., 2018). For example, *Collimonas* bacteria, although unable to use oxalate for growth, can be attracted by oxalate to sense and attack the fungal source (Rudnick et al., 2015). However, the specific involvement of oxalic acid from seed exudates in the microbe–plant interaction remains to be documented (Palmieri et al., 2019).

*Azorhizobium caulinodans* ORS571, a symbiont of the host *Sesbania rostrata*, can fix atmospheric nitrogen in free-living and symbiotic states. As reported for other rhizobia symbiotic associations (Caetano-Anolles et al., 1988; Merritt et al., 2007; Miller et al., 2007; Scharf et al., 2016), the establishment of the *A. caulinodans-S. rostrata* association involves chemotaxis toward plants roots, colonization of the root surface, infection, and the initiation of nodule organogenesis (Jiang et al., 2016; Liu et al., 2017, 2019a,b; Liu W. et al., 2018; Liu X. et al., 2018). In particular, two chemoreceptors, IcpB and TlpA1, were shown to be involved in the chemotactic response of *A. caulinodans* to some organic acids, such as succinate, citrate, tartrate, and malate (Jiang et al., 2016; Liu et al., 2017). IcpB, encoded by *icpB*, is a soluble heme-binding protein that is employed for aerotaxis and chemotaxis through sensing oxygen (Jiang et al., 2016). TlpA1 is a transmembrane chemoreceptor, encoded by *tlpA1*, that is located upstream of the chemotaxis gene cluster (*che* cluster containing *cheA*, *cheW*, *cheY1*, *cheB*, and *cheR* genes) in the *A. caulinodans* genome (Liu et al., 2017). Recently, we have identified TlpH, a transmembrane protein containing a dCache domain, as the cognate chemoreceptor of histidine, arginine, and aspartic acid, which are the three most abundant amino acids present in the seed exudates of *S. rostrata* (Liu et al., 2019a). In addition to their role in the chemotactic response of *A. caulinodans*, it was also shown that amino acids from seed exudates played an indirect role, being involved in the regulation of *che* genes and flagella synthesis (Liu et al., 2019a).

The objective of this work was to understand the contribution of organic acids from *S. rostrata* seed exudates in the process of *A. caulinodans* chemotaxis and root colonization. Characterization of the chemotactic response induced in *A. caulinodans* by the five most abundant organic acids present in *S. rostrata* seed exudates is reported. Among them, we focused on the role of oxalic acid in host colonization, its involvement as a chemoeffector, and its role in controlling chemotactic responses towards other chemoeffectors.

## Results

### Qualitative and Quantitative Analysis of Organic Acids in *S. rostrata* Seed Exudates

**FIGURE 1:**
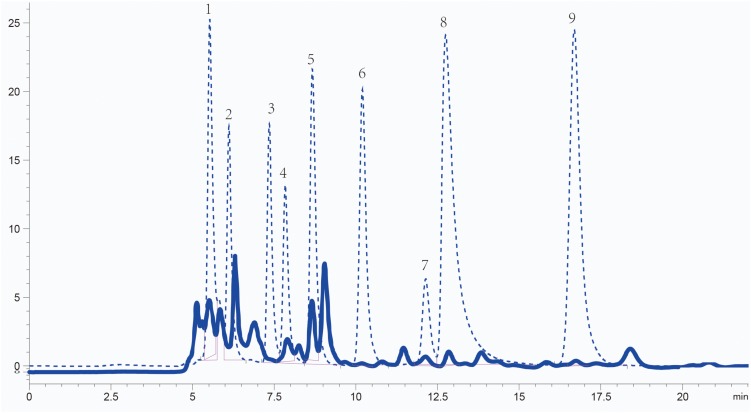
Identification of organic acids in *S. rostrata* seed exudates detected by HPLC. Elution peaks of organic acids from the exudates and standard samples are shown as solid and dashed lines, respectively. Numbers 1–9: oxalic acid, tartaric acid, malic acid, lactic acid, acetic acid, citric acid, succinic acid, maleic acid, and fumaric acid, respectively.

The concentration and composition of organic acids in *Sesbania* seed exudates were analyzed using high-performance liquid chromatography (HPLC). The organic acids present were identified by comparing their retention times with standard samples, and their concentrations were quantified according to the standard curve of standard substances. The five most abundant organic acids were acetic acid (1.20 × 10^3^ μM), succinic acid (3.62 × 10^2^ μM), lactic acid (2.78 × 10^2^μM), oxalic acid (4.43 × 10^1^ μM), and citric acid (7.97 μM). Maleic acid (1.89 μM) and fumaric acid (3.22 × 10^–1^ μM) were less abundant (Figure 1). Although malic acid and tartaric acid are reported as common in root or seed exudates (Kamilova et al., 2006; Tan et al., 2013), their content in *Sesbania* seed exudates was below the detection limit. The five most abundant organic acids in seed exudates were selected for further analysis.

### Effects of Organic Acids on the Growth of *A. caulinodans*

**FIGURE 2:**
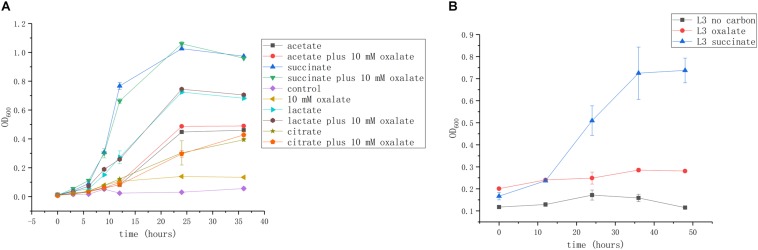
Growth of *A. caulinodans* ORS571 in liquid **(A)** and on soft agar plate **(B)** with each organic acid or plus oxalate as a carbon source. Values are means ± standard deviation from at least three experiments.

We next investigated whether these organic acids can be used as carbon and energy sources. Succinate is commonly used as a growth substrate in *A. caulinodans* growth medium (L3 minimal medium; see section “Materials and Methods”). Acetate, lactate, and citrate (10 mM) could support cell growth up to OD_600_ values of 0.5, 0.7, and 0.45, respectively. Though oxalate is abundant in seed exudates, it cannot promote *A. caulinodans* growth in liquid media (Figure 2A). Because the metabolic properties of substances in bacteria might be different between liquid and solid media, such as in the bacteria of the genus *Collimonas* (Fritsche et al., 2014), the growth curve of *A. caulinodans* with oxalate on semi-solid plates was also determined. Consistent with the result obtained in liquid cultures, oxalate cannot support growth on semi-solid plates (Figure 2B).

### Chemotactic Response of *A. caulinodans* ORS571 Toward Different Organic Acids

Bacterial chemotactic response toward organic acids was assessed using capillary and soft agar plate assays, using the wild type ORS571 and its derivate *cheZ* mutant, which does not display chemotactic response (Liu X. et al., 2018).

**FIGURE 3:**
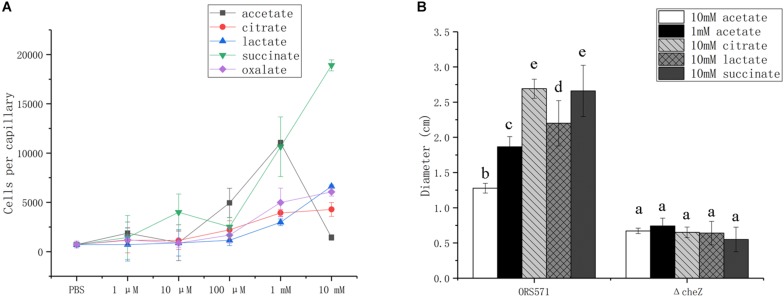
Chemotactic response of wild type and *cheZ* mutant strain toward different organic acids using capillary assay **(A)** and soft agar plate assay **(B)**. The same letter above the error bars indicates no significant differences between them according to a Duncan’s test. Error bars represent means ± standard deviation of three independent experiments.

In capillary assays, the chemotactic response of the wild type toward oxalate, citrate, and lactate was dose-dependent from 1 μM to 10 mM, while the response toward succinate was biphasic, with attractive concentration maxima at 10 μM and more than 1 mM; response to acetate followed a bell curve, with maximum attractive concentration at around 1 mM (Figure 3A).

In soft agar plate assay, the wild type showed chemotactic response to all of the organic acids assayed, while no response was detected with the *cheZ* mutant used as negative control (Figure 3B). Although *A. caulinodans* cells grow better with 10 mM than with 1 mM acetate (data not shown), 10 mM is not a suitable concentration for attractive response in capillary assay. Therefore, 1 mM acetate was also used to explore chemotactic response on the soft agar plate. The chemotactic rings formed by cells with 1 mM acetate are much larger than those with 10 mM acetate (Figure 3B), consistent with the results in the capillary assay.

### The Transmembrane Chemotaxis Receptor TlpA1 Is Involved in Chemotactic Response to Oxalate

**FIGURE 4:**
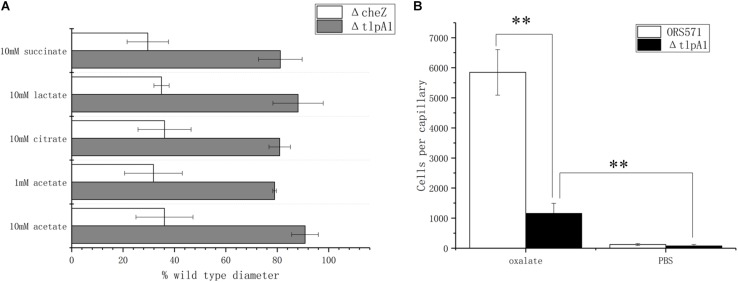
Role of TlpA1 in the chemotactic response of *A. caulinodans* ORS571 toward different organic acids using soft agar plate assay **(A)** and capillary assay **(B)**. Asterisks (^∗∗^*P* < 0.01) show a significant difference between conditions according to a *t*-test. Error bars are means ± standard deviation from at least three experiments.

It was previously shown that deletion of the *tlpA1* gene, which encodes the transmembrane chemotaxis receptor TlpA1, impaired the chemotactic response toward a broad range of ligands including tartrate, malate, proline, and glycerol (Liu et al., 2017) as well as succinate and citrate, identified as major organic acid components in seed exudates in the present work. Using soft agar plates, it was found that the Δ*tlpA1* mutant strain was also defective in chemotaxis toward lactate and acetate (Figure 4A). However, as the chemotactic rings were reduced but not abolished, it is likely that other unidentified chemoreceptors are involved in the chemotactic response toward these organic acids. In the case of oxalate, as this substrate is not used for growth, it cannot be assayed using the soft agar plate system. This is why the capillary assay was used instead. Indeed, Δ*tlpA1* was significantly defective in chemotactic response to oxalate, suggesting that TlpA1 is the cognate chemoreceptor to oxalate (Figure 4B).

### Oxalate Affects the Chemotactic Response to Different Organic Acids

Recently, we have found that aspartate, histidine, and arginine in seed exudates have indirect effects promoting the chemotactic response to succinate (Liu et al., 2019a). Therefore, we investigated whether oxalate also has an indirect effect on response to other organic acids using the soft agar plate assay. Indeed, as ORS571 cannot use oxalate as a growth substrate (Figures 2A,B), its concentration remained unchanged in soft agar plates.

**FIGURE 5:**
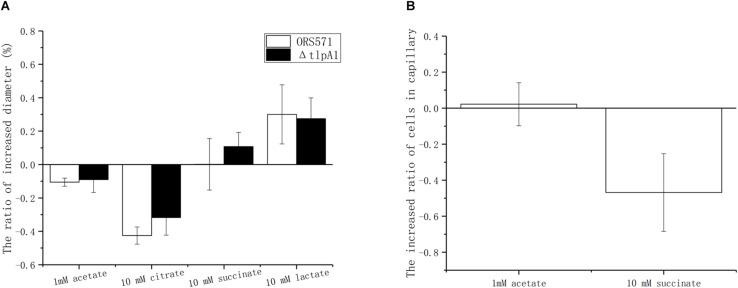
Effect of 10 mM oxalate on the chemotactic response of *A. caulinodans* ORS571 to other organic acids using soft agar plate assay **(A)** and capillary assay **(B)**. Data are expressed as means ± standard deviation from at least three experiments.

Chemotactic ring diameters on soft agar plates were compared using either a single organic acid (succinate, citrate, acetate, or lactate) or the same organic acid as a carbon source plus oxalate. When 10 mM oxalate was added, the chemotactic response toward acetate and citrate decreased by around 10 and 40%, respectively, while chemotaxis toward lactate increased by about 25%. However, the chemotaxis toward succinate remained unchanged. Changes observed using the Δ*tlpA1* mutant strain were similar to those with the wild type when oxalate was added (Figure 5A).

The role of oxalate was then assayed using the capillary method. There were two types of treatments. In control groups, the cells in the wells were suspended with phosphate-buffered saline (PBS buffer) only. In the experimental group, cells in wells were suspended with PBS buffer already containing 10 mM oxalate to ensure that there were no gradients of oxalate between the solutions in wells and in capillaries. Compared with the control group, adding oxalate decreased the chemotaxis toward succinate by about 56%, while the chemotactic response to 1 mM acetate remained unchanged (Figure 5B). Unfortunately, in the presence of oxalate, the chemotactic response to lactate and citrate varied from one experiment to another so that no conclusion could be formulated (data not shown).

### The Effect of Oxalate on the Chemotactic Response to Other Chemoeffectors Is Concentration-Dependent

The indirect effect of oxalate on the chemotactic response differs depending on the organic acids used (Figure 5A). Experiments were repeated in order to find out whether the oxalate effect on each of the organic acids was concentration-dependent. Oxalate concentrations from 0 to 10 mM were used. In control experiments, the relative diameter of the chemotactic ring formed on soft agar plates induced by each of the organic acids (succinate, citrate, acetate, and lactate) in the absence of oxalate was normalized to 1. The relative diameters on a soft agar plate with each organic acid and different concentrations of oxalate were then compared with their respective controls.

**FIGURE 6:**
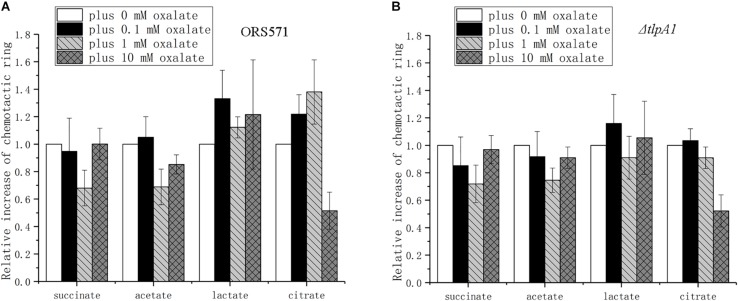
Role of oxalate at different concentrations in the chemotactic response of wild type and Δ*tlpA1* strains toward other organic acids using soft agar plate assay. The diameter of chemotactic rings with different organic acids was normalized by reference to chemotactic rings obtained in the absence of oxalate. Wild type **(A)** and Δ*tlpA1* **(B)**. Error bars represent means ± standard deviation from at least three experiments.

The chemotactic response to different organic acids was found to depend on the oxalate concentration used. Chemotaxis toward succinate presented a trend of first decreasing and then increasing to the original level as the concentration of oxalate increased (Figure 6A). The changes for acetate were similar to those for succinate, but the diameter of its chemotactic rings did not reach the original level. The chemotactic response to lactate showed a trend of first increasing and then decreasing to a higher level than the original level. The chemotactic response to citrate was similar to the response to lactate, but it decreased to a lower level than the original level. Compared with the wild type, Δ*tlpA1* showed different changes in chemotaxis toward citrate plus different concentrations of oxalate, while for the chemotactic response to succinate, acetate, and lactate, there were no differences between the two strains (Figures 6A,B).

### Oxalate Affects *A. caulinodans* Root Colonization Properties

**FIGURE 7:**
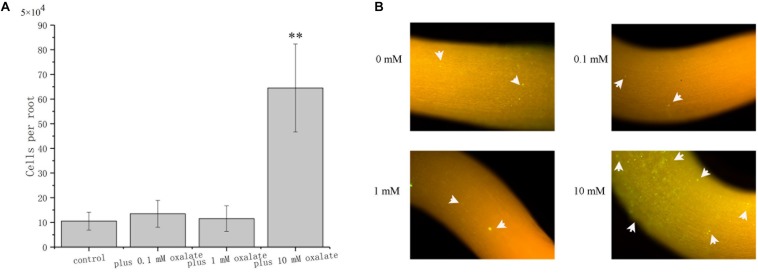
Role of oxalate at different concentrations in the colonization of *S. rostrata* roots. Representative photographs showing the colonization of ORS571-eGFP **(A)**. Quantitative comparison of the colonization of *A. caulinodans* **(B)**. Asterisks (^∗∗^*P* < 0.01) indicate a significant difference from control according to a *t-*test. These experiments were performed at least three times. Values are means ± standard deviation from at least three experiments.

The role of oxalate on root surface colonization was determined by evaluating the colonization properties of *A. caulinodans* ORS571 at different concentrations of oxalate. After incubating for 1 h, bacterial cells colonizing the surface of *S. rostrata* root were counted. Oxalate at 0.1 and 1 mM did not affect the extent of root colonization by ORS571, while oxalate at 10 mM, which has a strong chemo-attractive effect, increased root colonization significantly more than 6-fold (Figure 7A). This result was confirmed by qualitative investigation of colonization using GFP marked strain ORS571-GFP. In the presence of 10 mM oxalate, colonies with green fluorescence can be observed on the surface of roots. Consistent with the quantitative result, there was no apparent green fluorescence on the root surface of seedlings when 0, 0.1, or 1 mM oxalate was added (Figure 7B).

## Discussion

In this work, we explored the role of organic acids from *Sesbania* seed exudates on the interaction between *S. rostrata* and *A. caulinodans*. The results showed that *A. caulinodans* ORS571 could grow in the presence of citric acid, acetic acid, and lactic acid but could not grow in the presence of oxalic acid, indicating that the majority of the most abundant organic acids in exudates could serve as sole carbon resources. In addition, all the five most abundant organic acids in seed exudates could induce a strong chemotactic response, though their chemotactic strength at different concentrations was significantly different. Succinic acid chemotactic response showed a biphasic curve in capillary assay, indicating that succinic acid could function both at lower and higher concentrations. Compared with the other organic acids, succinic acid induced the strongest chemotactic response, showing that it might be an important signal molecule in seed exudates. In addition, oxalic acid is unique among the five organic acids. It is present at high concentration in exudates and could induce a strong chemotactic response, but it cannot be used to support growth.

In *A. caulinodans*, 43 genes encoding putative chemoreceptors were identified throughout the genome. Among them, TlpA1 has been reported to be a broad-range chemoreceptor. Chemoreceptors containing a broad range of ligands are more likely involved in the plant–microbe interactions. For example, deletion of genes encoding McpA and McpC possessing a broad range of ligands in *B. amyloliquefaciens* impaired the attraction toward cucumber exudates (Feng et al., 2018a, b). A significant decrease in colonization of *Arabidopsis thaliana* was observed in *Bacillus subtilis* mutants in which two chemoreceptor genes for amino acids, *mcpB* and *mcpC*, were inactivated (Allard-Massicotte et al., 2016). TlpA1 was previously shown to be involved in sensing some organic acids, glycerol, and amino acid in *A. caulinodans* (Liu et al., 2017). In addition, the Δ*tlpA1* mutant was found to be impaired in competitive colonization and nodulation of *Sesbania* (Liu et al., 2017), which confirmed the role of broad-ligand range chemoreceptors in plant–microbe interactions. In this study, we further broadened the ligand range of TlpA1, including three of the five most abundant organic acids secreted in seed exudates. Thus, the defect of the Δ*tlpA1* mutant strain in competitive colonization may result from the defect of chemotactic response to these organic acids. However, the deletion of *tlpA1* in *A. caulinodans* ORS571 resulted in a partial defect on the chemotactic response to organic acids and some other attractants, suggesting that other potential cognate chemoreceptors may be involved and that these chemoreceptors may contribute to the attraction toward the root surface collaboratively.

Oxalic acid and its conjugated base, oxalate, are found in various terrestrial environments including the rhizosphere of plants (Palmieri et al., 2019). They have been found in the exudates of many plants, such as tomato (de Weert et al., 2002; Tan et al., 2013), Barmultra grass (Kuiper et al., 2002), tobacco (Wu et al., 2015), banana (Yuan et al., 2015), and others. How can oxalic acid from exudates affect *A. caulinodans–S. rostrata* interactions? First, oxalic acid, as a chemoreceptor ligand, could induce a strong chemotactic response. Chemotactic behavior is a key factor for the colonization of hosts (Erhardt, 2016). As previously described, many organic acids from plant exudates can induce a chemotactic response and promote bacterial recruitment (de Weert et al., 2004; Tan et al., 2013). Second, oxalic acid regulates chemotactic behavior using indirect strategies as a potential signal affecting the chemotactic response of *A. caulinodans* to other stimuli. When oxalic acid was added on a soft agar plate, it affected the chemotactic response to different organic acids. In *Pseudomonas putida*, acetate could improve the transcript level of McpQ, whose cognate ligand is citrate, up to 20-fold (Lopez-Farfan et al., 2017), suggesting that the indirect role of some organic acids on chemoeffectors is not specific to *A. caulinodans*.

In this work, we further found that the indirect role of oxalic acid on *A. caulinodans* chemotactic response toward different organic acids depends on its concentration. At low concentrations, oxalic acid promotes the chemotactic response of bacteria toward organic acid, while it reduces the chemotactic response to them at higher concentrations. In other words, oxalic acid is a chemotaxis enhancer at low concentrations, while, at high concentrations, oxalic acid is a chemotaxis inhibitor.

What is the physiological significance of the concentration-dependent indirect role of oxalic acid on the building of *A. caulinodans–S. rostrata* associations?

First, the significance of the concentration-dependent effect of oxalic acid might result in promoting bacteria located at some distance to move toward plants quickly and in preventing the escape of bacteria from the spermosphere and rhizosphere areas. A concentration-dependent effect of maize root exudates on chemotaxis has also been reported, and exudates could promote the expression of chemoreceptors at a distance from plants (Lopez-Farfan et al., 2019). Second, oxalic acid might be a colonization enhancer of *A. caulinodans* at high concentrations. In general, adding an exogenous strong chemotactic attractant could impair the establishment of bacteria and host associations. For *Vibrio fischeri*, the efficiency of colonization of host squid could be diminished with exogenous addition of chemoattractant (GlcNAc)_2_, causing chemotactic gradient disruption (Mandel et al., 2012). For *H. pylori*, adding exogenous urea into the lumen of mouse stomach could also confound bacterial accumulation to damage sites, which can be detected by a urea chemoreceptor, TlpB (Hanyu et al., 2019). However, the strong chemotactic response induced by a low concentration of oxalic acid did not disturb *A. caulinodans* root colonization, while a high concentration of oxalic acid enhanced the colonization. These results indicate that the direct role of oxalic acid on chemotaxis might not be essential for colonization of *A. caulinodans*. Third, oxalic acid might be a switch that regulates movement and colonization on the plant surface. At low concentrations, oxalic acid promotes motility, while at high concentrations, oxalic acid enhances colonization. The second messenger c-di-GMP was shown to be a key factor affecting motility, chemotactic behavior, and biofilm formation, but Lopez-Farfan et al. (2019) established that c-di-GMP was not involved in chemotaxis promotion by exudates. At this stage, a possible role for oxalic acid on the c-di-GMP level cannot be excluded, even though we have failed to quantify c-di-GMP so far. It remains that a dual role of oxalic acid on motility and colonization is shown in this study. Since chemotaxis and biofilm formation both are prerequisites for colonization, we suspect that oxalic acid could also promote biofilm formation on abiotic surfaces. Unfortunately, calcium ions are essential for the biofilm formation of *A. caulinodans* (unpublished data), and the formation of calcium oxalate prevented us from exploring the potential role of oxalic acid on biofilm formation.

In this work, we established that the five most abundant organic acids, including oxalic acid, in *S. rostrata* seed exudates were involved in *A. caulinodans* chemotaxis and identified that TlpA1 was their cognate chemoreceptor. In addition, we determined that oxalic acid contributes a concentration-dependent effect on the chemotactic response of *A. caulinodans* ORS571 using several strategies. These results are important for understanding the role of organic acid on the bacteria–host interactions. Under gnotobiotic conditions, the relative concentration of oxalic acid resulting from seed and root exudation decreases depending on distance from the root system but may remain stable since it cannot be metabolized by *A. caulinodans*. However, in natural soil environments, the metabolism of oxalic acid by oxalotrophic bacteria could constitute a strong sink for oxalic acid and thereby enhance the formation of concentration gradients of oxalic acid in the rhizosphere (Canarini et al., 2019). Thus, the real role of oxalic acid on *A. caulinodans* and *S. rostrata* may be stronger and more complicated. Further work will explore the specific pathways or proteins regulated by oxalic acid and its effect on the microbial community.

## Materials and Methods

### Strains, Plasmids, and Media

All strains and plasmids used in this study were listed in Table 1. *A. caulinodans* ORS571 (Dreyfus et al., 1988) and its derivatives, Δ*cheZ* and Δ*tlpA1*, were grown in TY liquid media or in modified L3 minimal medium (containing 10 g/L DL sodium lactate as a carbon source and 10 mM NH_4_Cl as a nitrogen source) at 37°C, as previously described (Jiang et al., 2016). For growth and chemotaxis assays, in liquid or soft agar plates, the L3 minimal medium mineral base (devoid of carbon source) was supplemented with the carbon sources indicated in the text (see below). The concentrations of antibiotics used for *A. caulinodans* were as follows: 50 μg ml^–1^ kanamycin, 100 μg ml^–1^ ampicillin, and 50 μg ml^–1^ gentamycin.

**TABLE 1:** Bacteria strains and plasmids used in this study.

**Strain or plasmid**	**Relevant characteristics^a^**	**Source or references**
**Strains**		
*E. coli*		
DH5α	F- *supE44 AlacU169 (?80 lacZ*Δ*M15) hsdR17 recA1 endA1 gyrA96 thi-1 relA1*	Transgen
***Azorhizobium caulinodans***		
ORS571	Type strain; Am^R^, Na^R^	Dreyfus et al., 1988
ORS571-eGFP	Type strain; Am^R^, Na^R^, Km^R^	This study
Δ*cheZ*	ORS571 derivative; *cheZ* deletrion mutant, Am^R^, Na^R^, Gm^R^	Liu X. et al., 2018
Δ*tlpA1*	ORS571 derivative, *tlpA1* deletion mutant; Am^R^, Na^R^, Gm^R^	Liu et al., 2017
Plasmids		
pBBR-eGFP	Fluorescence mark plasmid, Km^R^	This study
pRK2013	Helper plasmid, ColE1 replicon; Tc^R^ + Km^R^	Ditta et al., 1980
pBBR1MCS-2	Broad-host-range plasmid; Km^R^	Kovach et al., 1995

*Am^*R*^, ampicillin resistance; Gm^*R*^, gentamycin resistance; Km^*R*^, kanamycin resistance; Na^*R*^, nalidixic acid; Tc^*R*^, tetracycline.*

### Construction of eGFP-Marked Strains

To construct a fluorescent marked *A. caulinodans*, the egfp gene from pEGFP-N1 (Mountain View, CA, United States) was amplified by PCR using one primer pair (gfp-*Sal*I-F: GTCGACATGGTGAGCAAGGGCGAG, and gfp-*Xba*I-R: TCTAGA TTACTTGTACAGCTCGTC). The amplicon and a broad host plasmid pBBR1MCS-2 (Kovach et al., 1995) were then digested with restriction enzymes (*Sal*I and *Xba*I) and linked together to generate pBBR-eGFP. The pBBR-eGFP was next introduced into ORS571 by triparental conjugation using helper plasmid pRK2013 (Ditta et al., 1980), using kanamycin as a selective marker, and the fluorescence of the resulting transconjugant strain ORS571-eGFP was verified by microscopy.

### Seed Sterilization and Exudate Collection

*Sesbania rostrata* seeds (40 g) were treated with sulfuric acid to sterilize the surface, as previously described (Liu et al., 2019a). After being soaked in the concentrated sulfuric acid for 30 min, seeds were washed with sterilized water at least five times and transferred to an incubator at 37°C for 48 h. After 48 h, 100 ml of sterile water was added to the seeds to collect the exudates. This was performed two times. The liquid phase containing the exudates was then recovered and used for the analysis of organic acids.

### Analysis of Organic Acids in Seed Exudates

An HPLC AQ-_C_18 (4.6 × 250 μm, Agilent, Santa Clara, CA, United States) was used to analyze the low molecular weight organic acids. The mobile phase consisted of solutions of 10 mmol L^–1^ K_2_HPO_4_, at pH 2.55, with a gradient elution of 0.5 mL min^–1^. Peak detection was performed with an ultraviolet (UV) detector at 210 nm. Nine organic acids that are common in plant exudates, namely oxalic acid, tartaric acid, malic acid, lactic acid, acetic acid, citric acid, succinic acid, maleic acid, and fumaric acid, were used as standards. Ten microliter exudate samples and standards were sequentially injected into the chromatographic system under the same running conditions. Each sample was analyzed at least three times.

### Growth of *A. caulinodans* on Organic Acids as the Carbon Source

*Azorhizobium caulinodans* cells were cultured in TY medium overnight. Cells were collected and washed with L3 minimal medium without a carbon source at least three times. The cell suspension was then inoculated into L3 minimal medium containing the desired source of organic acid at the concentration indicated in the text and adjusted at pH 7.2, at an initial OD_600_ of 0.01. Culture OD was determined sequentially over 36 h. The determination of growth of *A. caulinodans* ORS571 on soft agar plates (0.3%) with oxalic acid as a sole carbon source was made according to Rudnick et al. (2015). Plates were incubated at 37°C. At 0, 12, 24, 36, and 48 h, 2 ml of 1 g/L NaCl was added into each plate to swirl and collect the suspended bacteria, and the OD_600_ of the suspension was determined. More than five plates were inoculated as necessary for OD determination.

### Quantitative Capillary Chemotaxis Assay

For capillary assay, cell suspensions adjusted to an OD_600_ of 0.01 were inoculated into a 96-well plate, as described previously (Liu X. et al., 2018). Capillaries (Microcaps, Dummond Scientific) that were open at one end were inserted into the desired chemoattractant solution at the concentrations indicated in the text and then inserted into the well plates at 37°C for 1 h, as described in Liu X. et al. (2018). Capillaries filled with PBS were used as control. The liquid in the capillaries was then pipetted into 1 ml PBS and, after serial dilution, plated on TY solid medium for counting of colony-forming units (CFUs). The experiments were repeated at least three times.

The role of oxalic acid on the chemotactic response to other organic acids was determined by adding oxalic acid into PBS and adjusting the pH to 7.2. This solution was either used for bacterial suspension or supplemented by other organic acids, as indicated in the text.

### Soft Agar Plate Chemotaxis Assay

For the soft agar plate assay, overnight cultures were collected and washed with L3 minimal medium devoid of a carbon source. Then, 5 ml of bacterial cell suspension adjusted to an OD_600_ of 0.6 were dropped into the center of soft agar plates (0.3% agar) containing the desired organic acids (succinic acid, oxalic acid, acetic acid, lactic acid, and citric acid) as the substrate. When exploring the role of oxalic acid in chemotaxis, the oxalic acid was added onto the soft agar plate and was then mixed uniformly. After culturing at 37°C for 48 h, the diameter of each chemotactic ring was recorded.

### *Sesbania* Seedling Colonization Assay

Seeds sterilized as described above were germinated and grown under sterile conditions for 3 days to obtain seedlings. Overnight cultures of *A. caulinodans* were washed three times and adjusted to an OD_600_ of 0.01. Seedlings were immersed in L3 minimal base medium with different concentrations of oxalic acid (0, 0.1, 1, and 10 mM) (as oxalate was not used) for 1 h at 37°C. Seedlings were then removed and washed with sterilized water at least five times. The roots were cut off and vortexed to recover the attached bacteria. One milliliter of sterilized water was added to suspend the cells from the vortexed root surface, and serial dilutions were plated onto TY solid medium supplemented with antibiotics. After culturing for 48 h at 37°C, the CFUs were counted and analyzed.

### Microscopy

Qualitative observations of seedlings colonized with *A. caulinodans* ORS571-eGFP were performed using a fluorescence microscope Olympus BX53 system microscope and recorded by an Olympus DP73 digital camera at ×10 magnification. The fluorescence signal was detected using a green fluorescent protein (eGFP) filter set. Images were taken under the same conditions.

### Statistical Analysis

Mean and standard errors were computed based on experiments in this work repeated at least three times. A *t*-test (*P* < 0.01) was used to determine significant differences from control in capillary assay and colonization assay. A Duncan’s test (*P* < 0.01) was used for multiple comparisons. SPSS version 20 (IBM Corp., Armonk, NY, United States) was employed to perform analysis of variance (ANOVA) and various tests.

## Data Availability Statement

This manuscript contains previously unpublished data. The name of the repository and accession number(s) are not available.

## Author Contributions

XL and ZX conceived and designed the experiments, analyzed the data, prepared the figures and tables, and wrote the manuscript. XL, KZ, and YL carried out the experiments. CZ helped with the improvement and revision of the manuscript. All authors approved the submitted manuscript for publication.

## Conflict of Interest

The authors declare that the research was conducted in the absence of any commercial or financial relationships that could be construed as a potential conflict of interest.
